# Oral Manifestations of Human Immunodeficiency Virus-Infected Patients 

**Published:** 2015-01

**Authors:** Atessa Pakfetrat, Farnaz Falaki, Zahra Delavarian, Zohreh Dalirsani, Majid Sanatkhani, Mahsa Zabihi Marani

**Affiliations:** 1 *Oral and Maxillofacial Diseases Research Center, School of Dentistry, Mashhad University of Medical Sciences, Mashhad, Iran.*; 2 *Dental Research Center, School of Dentistry, Mashhad University of Medical Sciences, Mashhad, Iran.*; 3 *General Dentist, Mashhad, Iran.*

**Keywords:** Acquired Immunodeficiency Syndrome, HIV, Oral manifestations

## Abstract

**Introduction:**

Oral lesions are among the earliest clinical manifestations of human immunodeficiency (HIV) infection and are important in early diagnosis and for monitoring the progression to acquired immunodeficiency syndrome (AIDS). The purpose of this study was to determine the prevalence of oral lesions and their relationship with a number of factors in HIV/AIDS patients attending an HIV center.

**Materials and Methods:**

A total of 110 HIV-positive patients were examined to investigate the prevalence of oral lesions according to the criteria established by the European Community Clearing House on Oral Problems Related to HIV Infection. An independent T-test was used for correlation of oral lesions with CD4+ count and a χ2 test was used for analysis of the relationship of co-infection with hepatitis B virus (HBV), sexual contact, route of transmission, history of drug abuse, and history of incarceration.

**Results:**

Most of the cases were male patients (82.7%). The mean age across all participants was 36.2±8.1 years. Rampant carries, severe periodontitis and oral candidiasis were the most notable oral lesions. Oral lesions were more prevalent in patients between 26–35 years of age. There was a significant difference between patients with and without pseudomembranous candidiasis and angular cheilitis according to mean level of CD4+.

**Conclusion:**

The most common oral presentations were severe periodontitis, pseudomembranous candidiasis and xerostomia.

## Introduction

Human immunodeficiency virus (HIV)/ acquired immunodeficiency syndrome (AIDS) is currently the fourth leading cause of death worldwide. Because of its nature as a socio-psycho-economical problem, HIV infection and AIDS is one of the major threats in life. Since 1981 when HIV infection was first described, different oral conditions associated with HIV/AIDS have been defined. Although two laboratory markers, CD4+ lymphocyte count and HIV viral load, are important for monitoring HIV disease progression, these tests are not available in many developing countries. Therefore clinical findings are used in the screening of the disease process. Because of the ready accessibility of the oral cavity and because oral manifestations are the earliest and most important clinical indicators of HIV infection, researchers tend to focus on oral lesions. Oral findings play an important role in the detection of infection, prediction of viral infection progress and progression to AIDS. 

According to the result of various studies, oral lesions are observed in 70–90% of HIV-positive patients during the different stages of the disease (1-3). 

These lesions include oral candidiasis, hairy leukoplakia, Kaposi sarcoma, linear gingival erythema, necrotizing ulcerative periodontitis, aphthous ulcer (4,5). Other lesions reported in some articles are human papillomavirus infection,hyperpigmentation, oral submucous fibrosis, xerostomia, leukoplakia, herpes zoster, non-Hodgkin’s lymphoma, histoplasmosis, carcinoma, penicilliosis marneffei, exfoliative cheilitis, HIV salivary gland disease, perioral molluscum contagiosum, staphylococcus aureus infections, and petechiae (4,6). 

Oral lesions affect the quality of these patients’ lives and are strongly associated with the psychological health of patients in society. The number of HIV-infected patients in Iran is increasing, and by September 2012 had reached 25,041 people. Furthermore, among these cases, the infection had progressed to AIDS in 3,746 patients (7). In December 2011, there were approximately 500 HIV^+^ patients in Khorasan Razavi (8). Since Khorasan Province is one of the most crowded provinces in Iran, and because a comprehensive survey of the prevalence of oral lesions has not yet been performed in Khorasan Province, this study was designed to evaluate oral lesions in HIV/AIDS patients referred to the HIV Clinic or Mosallasi Center of Khorasan between 2008 and 2010. 

Various studies have reported different results concerning type and percentage of oral lesions. It appears that race, sex, route of transmission and stage of disease progression could affect the development and presence of oral findings. Because the oral manifestation of HIV/AIDS has not been studied in North- Eastern Iran, the aim of the present study was to determine the prevalence of oral lesions in HIV/AIDS patients attending the HIV center in Mashhad and its relationship with a number of important factors.

## Materials and Methods

In this cross-sectional study, HIV/AIDS patients at the HIV Clinic of the Infection and Behavioral Diseases Consulting Center underwent oral examination over a 15-month period from 2008 to 2010. The infection of all patients was confirmed using Enzyme-Linked Immunosorbent Assay (ELISA) and Western Blot tests, and all patients agreed to cooperate and sign a verbal agreement informed consent. 

A total of 110 HIV-positive patients enrolled in this study. Classification of HIV/AIDS patients was based on CDC disease staging system. The Center for Disease Control and Prevention (CDC) defined a set of guidelines for HIV-infected patients on the basis of clinical conditions associated with the HIV infection and CD4+ T-lymphocyte counts. 

Initially, the medical documents of all patients were evaluated and the demographic information of patients, the diagnosis date of infection by laboratory tests, the disease stage in the first examination, history of incarcerations, needle stick, sexual contacts, history of addiction, use of methadone, co-infection with other viruses such as hepatitis B virus (HBV), hepatitis C virus (HCV) and human T-lymphotropic virus type-1 (HTLV1), and CD4+ cell count at the time of oral examination were recorded. Next, the patients underwent precise dental, oral, and periodontal examinations using a periodon- tal probe, mouth mirror, and dental light in a dental chair. 

Oral mucosa examination and dental and periodontal evaluation was performed by an experienced dentistry student and confirmed by two oral medicine specialists and a periodontist familiar with HIV oral lesions, respectively. The system used for classification of oral lesions was the European Community (EC) Clearinghouse on Oral Problems related to HIV Infection and WHO Collaborating Center on Oral Manifestations of the Human Immunodeficiency Virus, 1993 (9). 

The Periodontal Disease Index (PDI) and Periodontal Index (PI) were used as diagnostic criteria for evaluation of periodontal diseases. The examiners also provided complementary tests and treatments in some cases, if required. The study protocol was approved by the Institutional Ethics Committee (IEC) of Mashhad University of Medical Sciences. An independent T-test was used for analysis of the relationship of oral lesions with CD4+ count and a χ^2 ^test was used for analysis of the relationship of co-infection with HBV, sexual contacts, route of transmission, history of drug abuse, and history of incarceration. A P<0.5 was considered to indicate statistical significance.

## Results

The study population included HIV/AIDS patients with a mean age of 36.2 ±8.1 years. Among them, 82.7% were male with an average age of 36.6±7.9 years and the rest

were female patients with average age of 34.1±9.1 years. The most common oral problem in all patients was tooth decays (41.8%). 

CD4+ count was greater than 200 in all patients, and all patients were asymptomatic or reported mild symptoms (Stage I–II disease). The most common oral mucosal findings in both sexes was severe periodontitis (30%), pseudomembranous candidiasis (26%) and hairy tongue (26%) (Table.1). 

A number of lesions, including angular cheilitis (P=0.046), verruca vulgaris (P=0.004), and fissured tongue (P=0.029), were significantly more common in women. Table 1 shows the distribution of oral lesions according to gender. Evaluation of lesion prevalence according to age revealed a significant increase in the prevalence of herpes simplex virus (HSV) infection in patients over 45 years of age (P=0.05). The distribution of oral lesions based on age is shown in (Table. 1).

Evaluation of patients according to marital status revealed that the most common oral lesions among single patients were xerostomia (30.6%) and severe periodontitis (26.5%). In married patients, severe periodon titis (32.3%) and pseudo- membranous candidiasis (29%) were most common, while pseudomembranous candidiasis and herpes simplex infection (25%) were most prevalent in divorced patients, and HSV infection (60%) in widow/widowers. However, there was no significant difference in the prevalence of oral lesions in relation to marital status (P>0.05 for all lesions).

Furthermore, there was no significant difference in the distribution of oral lesions between patients living in urban and rural areas and the homeless (P>0.05). The most common oral presentations in all three groups were severe periodontitis, pseudomembranous candidiasis, and xerostomia, respectively. 

**Table 1:** Distribution of oral lesions according to age and gender

**Oral Findings**	**Total**	**>45 yrs**	**36-45yrs**	**26-35yrs**	**≥** ** 25yrs**	**P value**	**Female**	**Male**	**P value**
	No (%)	No (%)	No (%)	No (%)	No (%)		No (%)	No (%)	
Tooth Decays	46(41.8)	8(50)	14(37.1)	21(45.7)	3(42.9)	0.83	9(47.4)	37(40.7)	0.59
Severe Periodontitis	30(27.3)	6(37.5)	6(16.2)	15(32.6)	3(42.9)	0.21	6(31.6)	24(26.4)	0.64
Pseudomembranous Candidiasis	26(23.6)	5(31.2)	8(21.6)	13(28.3)	0(0)	0.36	5(26.3)	21(23.1)	0.76
Hairy Tongue	26(23.6)	5(31.2)	7(18.9)	13(28.3)	0(0)	0.29	3(15.8)	23(25.3)	0.37
Xerostomia	22(20)	5(31.2)	4(10.8)	13(28.3)	0(0)	0.08	4(21.1)	18(19.8)	0.9
HSV Infection	22(20)	4(43.8)	4(10.8)	9(19.6)	1(14.3)	0.05	3(15.8)	19(20.9)	0.61
Erythematous Candidiasis	21(19.1)	7(25)	5(13.5)	11(23.9)	1(14.3)	0.61	2(10.5)	19(20.9)	0.29
Hyperpigmentation	20(18.2)	2(12.5)	6(16.2)	10(21.7)	2(28.6)	0.73	5(26.3)	15(16.5)	0.31
Aphthous Ulcer	12(10.9)	2(12.5)	4(10.8)	4(8.7)	2(28.6)	0.48	1(5.3)	11(12.1)	0.38
Angular Cheilitis	10(9.1)	2(12.5)	6(16.2)	1(2.2)	1(14.3)	0.15	4(21.1)	6(6.6)	0.046
Non-specific Ulcers	9(8.2)	0(0)	5(13.5)	4(8.7)	0(0)	0.34	1(5.3)	8(8.8)	0.61
Verruca Vulgaris	7(6.4)	1(6.2)	2(5.4)	3(6.5)	1(14.3)	0.85	4(21.1)	3(3.3)	0.004
Fissured Tongue	6(5.5)	1(6.2)	4(10.8)	0(0)	1(14.3)	0.13	3(15.8)	3(3.3)	0.029
Keratosis	5(4.5)	1(6.2)	1(2.7)	2(4.3)	1(14.3)	0.60	1(5.3)	4(4.4)	0.86
Salivary Gland Disease	5(4.5)	1(6.2)	1(2.7)	3(6.5)	0(0)	0.77	0(0)	5(5.5)	0.29
Lichenoid reactions	5(4.5)	0(0)	2(5.4)	1(2.2)	1(14.3)	0.34	0(0)	5(5.5)	0.29
Trigeminal Neuralgia	5(4.5)	2(12.5)	1(2.7)	1(2.2)	0(0)	0.41	3(15.8)	2(2.2)	0.01
Necrotizing Ulcerative Gingivitis	2(1.8)	0(0)	0(0)	2(4.3)	0(0)	0.44	0(0)	2(2.2)	0.51
Necrotizing Ulcerative Periodontitis	3(2.7)	0(0)	0(0)	2(4.3)	1(14.3)	0.15	0(0)	3(3.3)	0.42
Trombocytopenic Purpura	1(0.9)	0(0)	0(0)	1(2.2)	0(0)	0.72	0(0)	1(1.1)	0.64

Evaluation of lesions with respect to history of incarceration showed that pseudomembranous candidiasis (23.3%), melanotic hyperpigmentation (20%), and xerostomia (16.7%) were the most common oral lesions in people without a history of incarceration. Fissured tongue (27.5%) and pseudomembranous candidiasis (23.8%) were the most common oral lesions in people with imprisonment history. Furthermore, the prevalence of trigeminal neuralgia was significantly higher in patients with a history of incarceration (P=0.007). No statistically difference was observed in the prevalence of oral lesions between drug users and non-drug users except for thrombocytopenic purpura and papillomatous lesions, which were more frequent in non-drug users (P=0.03 and P=0.008, respectively). In both groups, tooth decay, severe periodontitis, hairy tongue, and pseudomembranous candidiasis were the most common oral problems. 

In patients taking methadone to overcome addiction, melanotic hyperpigmentation (36.8%), pseudomembranous candidiasis (26.3%), and severe periodontitis (26.3%) were the most common oral lesions after tooth decay (52.6%). The prevalence of verruca vulgaris (P=0.004), fissured tongue (P=0.029), angular cheilitis (P=0.04), melanotic hyperpigmentation (P=0.02), and trigeminal neuralgia (P=0.01) was significantly higher in patients taking methadone than others. 

The prevalence of erythematous candidiasis was significantly higher in patients with a history of sexual contacts compared with those without sexual contacts (P=0.04) (Table. 2). 

**Table 2:** Distribution of oral lesions regarding to sexual contact

**Oral Findings**	**Total**	**Sexual Contact**	**Non Sexual Contact**	**P value**
	No (%)	No (%)	No (%)	
Tooth Decays	46(41.8)	20(47.6)	26(38.2)	0.33
Severe Periodontitis	30(27.3)	12(28.6)	18(26.5)	0.81
Pseudomembranous Candidiasis	26(23.6)	11(26.6)	15(22.1)	0.62
Hairy Tongue	26(23.6)	7(16.7)	19(27.9)	0.17
Xerostomia	22(20)	8(19%)	14(20.60	0.84
HSV Infection	22(20)	9(21.4)	13(19.1)	0.76
Erythematous Candidiasis	21(19.1)	12(28.6)	9(13.2)	0.04
Hyperpigmentation	20(18.2)	9(21.4)	11(16.2)	0.48
Aphthous Ulcer	12(10.9)	4(9.5)	8(11.8)	0.71
Angular Cheilitis	10(9.1)	3(7.1)	7(10.3)	0.57
Non-specific Ulcers	9(8.2)	4(9.5)	5(7.4)	0.68
Verruca Vulgaris	7(6.4)	1(2.4)	6(8.8)	0.17
Fissured Tongue	6(5.5)	3(7.1)	3(4.4)	0.54
Keratosis	5(4.5)	3(7.1)	2(2.9)	0.30
Salivary Gland Disease	5(4.5)	1(2.4)	4(5.9)	0.39
Lichenoid reactions	5(4.5)	2(4.8)	3(4.4)	0.93
Trigeminal Neuralgia	5(4.5)	2(4.8)	3(4.4)	0.93
Necrotizing Ulcerative Gingivitis	2(1.8)	1(2.4)	1(1.5)	0.72
Necrotizing Ulcerative Periodontitis	3(2.7)	1(2.4)	2(2.9)	0.86
Trombocytopenic Purpura	1(0.9)	1(2.4)	0(0)	0.20

The most common oral problems in both homosexual and heterosexual patients were tooth decay, severe periodontitis, pseudomembranous, and erythematous candidiasis. In fact, no significant difference was observed between homosexuals and heterosexuals. The only exception was verruca vulgaris which was more frequent in patients without a history of heterosexual contact (P=0.05). 

Common lesions in patients with homosexual contact included tooth decay, periodontal diseases, pseudomembranous candidiasis, erythematous candidiasis, hairy tongue, xerostomia, melanotic hyperpigmentation, and keratosis.

Only two patients recalled a history of needle stick, and the most common oral lesions in these cases were pseudo- membranous candidiasis, angular cheilitis, unspecific ulcer, and verruca vulgaris. Although the number of cases was very small, significant differences were observed between angular cheilitis (P=0.04), nonspecific ulcers (P=0.02), and verruca vulgaris (P=0.01) in these patients compared with others. 

*Analysis of oral lesions according to CD4+ count*

 The CD4+ cell count was greater than 200 cells/mm^3 ^in all patients. Therefore, our patients were in stage I or II of disease. Statistical analysis showed that CD4+ count plays a crucial role in oral candidiasis. The mean level of CD4+ in patients with pseudomembranous candidiasis at the time of oral examination was 358±177.6 cells/mm^3^, while the count was 468±229.5 cells/mm^3^ in patients free of this lesion (P=0.02). The mean level of CD4+ was 207.06±4.2 and 325.8±35.7 cells/mm^3^ in patients with and without angular cheilitis, respectively (P=0.04) (Table. 3).

**Table 3:** Distribution of oral lesions regarding to CD4 count

**Oral Lesions**	CD4 (mean SD)		P value	t
	Positive	Negative		
Tooth Decays	174.4 4.05	294.5±4.6	0.12	1.56
Severe Periodontitis	194.5 3.99	231.3±4.58	0.184	1.3
Pseudomembranous Candidiasis	358.8 177.6	468.4 ±229.5	0.02	2.2
Hairy Tongue	243.9 4.4	217.1±42.4	0.994	0.008
Xerostomia	163.3 4.2	235.6±4.4	0.588	0.546
HSV Infection	168.2 3.8	233.05±4.5	0.315	1.1
Erythematous Candidiasis	422.9 3.8	447.1±4	0.626	0.491
Hyperpigmentation	208.6 4.2	226.5±4.46	0.706	0.381
Aphthous Ulcer	253.4 5.05	218.74±4.34	0.37	0.92
Angular Cheilitis	207.06±4.2	325.8±35.7	0.043	2.04
Non-specific Ulcers	210.08 4.03	224.3±4.4	0.58	0.573
Verruca Vulgaris	157.7 4.7	226.7±4.4	0.641	0.485
Fissured Tongue	216.5±4.3	236.6 6.56	0.55	0.247
Keratosis	174.02±4.04	225.16±4.44	0.645	0.491
Salivary Gland Disease	215.9±4.55	223.9±4.41	0.899	0.134
Lichenoid reactions	217.13±4.4	348.8±4.8	0.774	0.307
Trigeminal Neuralgia	119.9±3.8	226.2±4.4	0.315	1.1
Necrotizing Ulcerative Gingivitis	111.7±3.09	223.6±4.45	0.32	1.66
Necrotizing Ulcerative Periodontitis	174.45±4.35	224.4±4.42	0.951	0.068
Trombocytopenic Purpura	220.6±1.5	221.8±4.45	0.191	1.3

*Co-infection with HBV, HCV and HTLV1:*

No significant difference was observed in oral conditions in HBV-positive patients compared with others (P>0.05). However, in HCV-positive patients, a significant increase was found in the prevalence of pseudomembranous candidiasis (P=0.01), oral pigmentations (P=0.02), nonspecific ulcers (P=0.003), and lichenoid reactions (P=0.01). A difference was also observed in the prevalence of xerostomia (P=0.02) and tooth decay (P=0.04) in patients co-infected with HTLV1. The relationship between oral problems and co-infection with these viruses is shown in (Table. 4).

**Table4:** Distribution of oral findings regarding to co-infection with HBV, HCV and HTLV1

**Oral Findings**	**HCV Positive**	**HCV** **Negative**	**P value** **(HCV)**	**HBV Positive**	**HBV Negative**	**P value** **(HBV)**	**HTLV1 Positive**	**HTLV1 Negative**	**P value** **(HTLV)**
	No (%)	No (%)		N0 (%)	No (%)		No (%)	No (%)	
Tooth Decays	25(43.1)	21(40.4)	0.77	6(54.5)	40(40.4)	0.36	6(75)	40(39.2)	0.04
Severe Periodontitis	20(34.5)	10(19.2)	0.73	4(36.4)	26(26.3)	0.47	2(25)	28(27.5)	0.88
Pseudomembranous Candidiasis	21(36.2)	5(9.6)	0.01	3(27.3)	23(23.2)	0.76	1(12.5)	25(24.5)	0.44
Hairy Tongue	16(27.6)	10(19.2)	0.30	3(27.3)	23(23.2)	0.76	1(12.5)	25(24.5)	0.44
Xerostomia	11(19)	11(21.2)	0.77	1(9.1)	21(21.2)	0.34	4(50)	18(17.6)	0.02
HSV Infection	11(19)	11(21.2)	0.77	1(9.1)	21(21.2)	0.34	3(37.5)	19(18.6)	0.19
Erythematous Candidiasis	14(24.1)	7(13.5)	0.15	2(18.2)	19(19.2)	0.93	1(12.5)	20(19.6)	0.62
Hyperpigmentation	6(10.3)	14(26.9)	0.02	1(9.1)	19(19.2)	0.41	0(0)	20(19.6)	0.16
Aphthous Ulcer	7(12.1)	5(9.6)	0.68	1(9.1)	11(11.1)	0.83	1(12.5)	11(10.8)	0.88
Angular Cheilitis	7(12.1)	3(5.8)	0.25	1(9.1)	9(9.1)	1	0(0)	10(9.8)	0.35
Non-specific Ulcers	9(15.5)	0(0)	0.003	0(0)	9(9.1)	0.29	0(0)	9(8.8)	0.38
Verruca Vulgaris	2(3.4)	5(9.6)	0.18	0(0)	7(7.1)	0.36	1(12.5)	6(5.9)	0.46
Fissured Tongue	3(5.2)	3(5.8)	0.89	0(0)	6(6.1)	0.40	0(0)	6(5.9)	0.48
Keratosis	4(6.9)	1(1.9)	0.21	1(9.1)	4(4)	0.44	0(0)	5(4.9)	0.52
Salivary Gland Disease	3(5.2)	2(3.8)	0.73	0(0)	5(5.1)	0.44	0(0)	5(4.9)	0.52
Lichenoid reactions	0(0)	5(9.6)	0.01	0(0)	5(5.1)	0.44	0(0)	5(4.9)	0.52
Trigeminal Neuralgia	1(1.7)	4(7.7)	0.13	0(0)	5(5.1)	0.44	0(0)	5(4.9)	0.52
Necrotizing Ulcerative Gingivitis	2(3.4)	0(0)	0.17	0(0)	2(2)	0.63	0(0)	2(2)	0.68
Necrotizing Ulcerative Periodontitis	3(5.2)	0(0)	0.09	0(0)	3(3)	0.55	1(12.5)	2(2)	0.07
Trombocytopenic Purpura	0(0)	1(1.9)	0.28	0(0)	1(1)	0.73	0(0)	1(1)	0.77

## Discussion

Because a study has not previously been conducted in Iran evaluating oral lesions in HIV/AIDS, this study was designed for the assessment of oral lesions with a standard international questionnaire. Oral lesions are the earliest and most important indicators of AIDS disease progression and may develop in up to 50% of HIV-infected patients and in up to 80% of AIDS patients (6).

In our study, rampant carries, severe periodontitis and oral candidiasis were the most notable oral lesions. Oral lesions were more prevalent in patients between 26–35 years of age and there was a significant difference between patients with and without pseudomembranous candidiasis and angular cheilitis according to mean level of CD4+.

A review of studies performed in developed and developing countries showed that the prevalence of oral manifestations was 13–99% (in Kenya and Spain, respectively) (4). One study in Tehran revealed that 149 of 200 HIV+ patients had oral lesions (74.5%). The most common lesions were oral candidiasis, linear gingival erythema, and lymphadenopathy (10). These lesions may influence the quality of life of patients because oral health is related to physical and mental health (11), potentially causing dysphasia, difficulty in speech and swallowing and consequently weight loss and clinical deterioration (11). 

In our study, the majority of patients were men (82.7%), similar to the gender distribution reported in a number of other studies. Previously published studies have revealed considerable variation in the proportion of men and women with HIV and oral lesions. One study showed that 82% of HIV-positive were male patients (12,13), although this proportion was higher than that reported in other studies (-). Other studies reported that 88.8% and 90.7 % of HIV+ patients referred to a dental clinic were men (18, 19). In another study conducted in England, 78.6% of subjects were homosexual men with the mean age of 35.3 years (17), while in a study in Zimbabwe, the male: female ratio was 1:1 (20). In contrast, 74.5% of patients studied in Uganda were women, and oral lesions were recognized in 72% of the patients (21). Other studies conducted in Zambia, Zaire, and Thailand showed that most infected patients were female (-). Thus, it seems that cultural and economic conditions influence the gender and age distribution. 

In our study, the mean age of patients was 36.2 ±8.1 years; similar to other studies in which the mean age of the patients was within fourth decade of life (15,18). 

In our study, no significant difference was observed in the frequency of oral lesions with age and sex; however there was a significant increase in the prevalence of HSV infection in patients over 45 years of age, and some lesions including angular cheilitis, verruca vulgaris, and fissured tongue had a significant gender bias towards women.

In our study, the high prevalence of pseudomembranous candidiasis has been detected. Although candidiasis is one of the most common lesions in HIV/AIDS, its development increases with decrease in CD4+ count (26). 

Candidiasis was reported as the most common lesion in some studies, which is similar to our study. However, its prevalence is higher in a number of studies (15,18,20,21).

The three common presentations of oral candidiasis are pseudomembranous candidiasis, erythematous candidiasis and angular cheilitis. In our study, the prevalence of pseudomembranous candidiasis and erythematous candidiasis were 23.6% and 19.1%, respectively. Although the prevalence of pseudomembranous candidiasis was less than the prevalence reported in the study by Bendlick (52.5%), the prevalence of erythematous candidiasis was similar to the rate of erythematous candidiasis in that study (22.8%) (15).

One study compared the oral finding between HIV-positive and healthy persons referring to a dental clinic, and demonstrated that oral candidiasis, hairy leukoplakia, HIV gingivitis and HIV periodontitis were the most common lesions in HIV+ patients (19). Although Kaposi sarcoma, salivary gland disease, and cancrum oris were the most common lesions in Zimbabwe (20), non-Hodgkin’s lymphoma, atypical ulcers and necrotizing ulcerative periodontitis were the least common lesions in Uganda (21).

Candidiasis is typically defined as the most common lesion in most studies; however, its frequency varies among studies. A review study of HIV infection in developing countries revealed that candidiasis was the most common lesion with a prevalence of 15–80% and pseudomembranous-type lesions were associated with severe immunosuppression. The prevalence of hairy leukoplakia ranged from 0–26% (27). Another review study demonstrated that oral candidiasis was the most common lesion, with varying frequency (from 5% in Minnesota to 94% in Zaire), whereas oral hairy leukoplakia had a frequency ranging from 0–43% (4). Although in some studies, oral hairy leukoplakia (OHL), which is caused by Epstein-Barr virus, has been defined as one of the most common oral lesions after candidiasis (15, 28), it was not observed in our patients. This is presumably due to the association between OHL and CD4+ count (less than 200).

Although it is expected that bilateral hairy leukoplakia would be observed more commonly in immune-suppressed patients, in one study bilateral and unilateral hairy leukoplakia were reported in 35.6% and 9.9% of patients, respectively(15). 

The mode of transmission differed across various studies and could affect the type and prevalence of oral manifestations (27). In a study by Sen, heterosexual contact was the most common mode of transmission (88.3%) (29). In a study reported by Glick, approximately 70% of patients were homosexual/ bisexual, 18.7% were intravenous drug users, and there was no significant difference between homosexuals and heterosexuals according to oral problems, except for verruca vulgaris which was more frequent in patients without a history of heterosexual contact (18). Nittayananta’s study showed a significant association between the route of HIV transmission and the risk of oral lesions, while oral lesions also have a significant association with heterosexual mode of transmission (28). 

In the present study most patients were drug users. Drug abuse is the major route for the transmission of blood-borne infections in Iran. It seems that the cultural and socio-economical characteristics of patients affect the mode of transmission, and consequently the prevalence of some lesions. In present study, however, no statistically significant difference was observed in the prevalence of oral lesions between drug users and non-drug users, except for thrombocytopenic purpura and papillomatous lesions which were more frequent in non-drug users. 

Furthermore, and in contrast to most earlier studies, the present study evaluated patients according to their marital status. It was anticipated that marital status may influence the mode of transmission; however, in our study, there was no significant difference between single and married patients according to the prevalence of oral lesions. Moreover, in the present study, living environment (urban or rural residents and homeless patients) had no effect on type of oral lesions. The most common oral presentations in all groups were severe periodontitis, pseudo- membranous candidiasis and xerostomia. Living environment could influence the mode of transmission; for example, living in public houses and prisons could increase transmission of blood-borne infections. Although some lesions such as pseudomembranous candidiasis were common in both individuals with and without a history of imprisonment, trigeminal neuralgia was significantly higher in patients with a history of incarceration. 

Currently, CD4+ count is used as a marker to identify the progression of HIV and stage of immune suppression. Most studies emphasized the association between oral lesions and CD4+ count (especially below 200 cells/mm^3^), an indicator of disease progression (24). In Brazil, among AIDS patients with CD4+ cell counts <200 cells/mm^3^, 50.7% had oral candidiasis, particularly of the pseudomembranous form (26). Oral candidiasis is a predictor of reduction in CD4+ and AIDS progression (30).

Different studies showed an association between reduction in CD4+ count with the presence of oral Kaposi sarcoma, non-Hodgkin’s lymphoma and necrotizing ulcerative periodontitis (18, 31). 

One study in India revealed that candidiasis is a common lesion in patients with a mean CD4+ count of 212 /mm^3^, and hairy leukoplakia was presented in subjects with a mean CD4+ count of 97/ mm^3^. No case of Kaposi sarcoma was reported in that particular study (29). Another study showed that common lesions in HIV/AIDS patients (including hairy leukoplakia, necrotizing ulcerative periodontitis, xerostomia, Kaposi sarcoma, HSV, and major aphthous ulcers) were concurrent with a decrease in CD4+ count. Further, a CD4+ count <100/mm^3^ belonged to major aphthous ulcers, necrotizing ulcerative periodontitis, Kaposi sarcoma, and HSV ulcers (18).

In Shiboski’s study, hairy leukoplakia was directly related with both decrease in CD4+ count and smoking. It seems that smoking could influence the local immune response of the oral mucosa (32). Furthermore, another study showed a strong association between hairy leukoplakia and alcohol or smoking (33). These results were similar to the present study in which a lower CD4+ count was observed in patients with pseudomembranous candidiasis, angular cheilitis and fissured tongue (correlation observed with CD4+ count <300 cells/mm^3^).

Some HIV/AIDS patients present with several oral lesions. In Bendlick’s study, only 9.9% of the patients had no oral lesions and about 60% had more than one oral lesion (15). The number of oral lesions occurring concurrently had a direct relationship with CD4+ count and progression of disease. In one study, patients with three or more oral lesions had mean CD4+ <100. Further, presence of three oral lesions in a patient has a 100% positive predictive value for finding a CD4+ count <200 cells/mm^3^ among HIV+ patients (18).

The relationship of co-infection with HBV, HCV and HTLV1 in HIV+ patients with frequency of oral lesion was another aspect evaluated in our study. Co-infection with different blood-borne infections indicates the possibility of having high-risk behaviors. 

In the present study, there was a significant increase in the prevalence of some oral lesions in patients co-infected with HCV and HTLV1. Since HTLV-1 is more common in the Khorasan Province than in other regions of Iran, high-risk patients are evaluated for this infection in the clinic. 

In one study performed in Sari, Iran, of 80 HIV+ patients, 11.3% were co-infected with HBV, 33.8% with HCV, and 25% with HBV/HCV (34). One study conducted in England revealed that the prevalence of oral manifestations in both groups of patients taking and not taking antiretrovirals decreased over time (17).

One limitation of our study was the lack of any patient with CD4+ <200 or in the progressive stage of AIDS; therefore, we cannot evaluate oral lesions in these patients. Since most of our patients used methadone or analgesic drugs or retroviral drugs, it was not possible to determine whether or not oral lesions are related to their drugs. This was another limitation of our study. 

Briefly, variable sample size, stage of the disease, severity of suppression of immune system, use of different criteria and regional patterns of HIV infection could explain the difference in prevalence of oral lesions in the different investigations. Since most of our patients were drug users, some differences with other studies may be related to mode of transmission. The cultural properties of every region should be considered in the study of the prevalence of oral lesions associated with HIV/AIDS.

## Conclusion

The most common oral findings in both sexes were severe periodontitis, pseudo- membranous candidiasis and hairy tongue. There was a significant increase in the prevalence of HSV infection in patients over 45 years of age. There was a significant difference between patients with and without oral manifestations of HIV+ according to CD4+ count.
